# Target-Controlled Infusion with PSI- and ANI-Guided Sufentanil Versus Remifentanil in Remimazolam-Based Total Intravenous Anesthesia for Postoperative Analgesia and Recovery After Laparoscopic Subtotal Gastrectomy: A Randomized Controlled Study

**DOI:** 10.3390/jcm14248921

**Published:** 2025-12-17

**Authors:** Byongnam Jun, Young Chul Yoo, Sun Joon Bai, Hye Jung Shin, Jinmok Kim, Na Young Kim, Jiae Moon

**Affiliations:** 1 Department of Anesthesiology and Pain Medicine, Anesthesia and Pain Research Institute, Yonsei University College of Medicine, Seoul 03722, Republic of Korea; bnjun@yuhs.ac (B.J.); seaoyster@yuhs.ac (Y.C.Y.); kjm9689@yuhs.ac (J.K.); 2 Department of Anesthesiology and Pain Medicine, Bucheon St. Mary’s Hospital, College of Medicine, The Catholic University of Korea, Bucheon 14662, Republic of Korea; sjbae@yuhs.ac; 3 Biostatistics Collaboration Unit, Department of Research Affairs, Yonsei University College of Medicine, Seoul 03722, Republic of Korea; hjshin105@yuhs.ac

**Keywords:** target-controlled infusion, remimazolam, remifentanil, sufentanil, opioid consumption, analgesia, quality of recovery-40

## Abstract

**Background/Objectives:** Target-controlled infusion (TCI) with remifentanil or sufentanil provides stable and effective anesthesia. This randomized prospective trial investigated the comparative efficacy of TCI using sufentanil versus remifentanil on postoperative analgesia and recovery profiles in patients after laparoscopic subtotal gastrectomy under remimazolam-based total intravenous anesthesia (TIVA). **Methods:** Sixty-six patients who underwent laparoscopic subtotal gastrectomy were randomly allocated to receive either TCI-based sufentanil or remifentanil in TIVA with remimazolam. The primary endpoint was the cumulative fentanyl consumption within 24 h after surgery. The secondary outcomes were pain intensity at rest and during activity, and recovery parameters including time to extubation, length of post-anesthesia care unit (PACU) stay, and quality of recovery (QoR-40) on postoperative day 1 (POD1). **Results:** The cumulative fentanyl consumption over the 24 h postoperative period was similar between the two groups. However, compared with the remifentanil group, the sufentanil group required significantly less fentanyl during the immediate postoperative period (0–0.5 h) (*p* < 0.001) and exhibited lower pain scores both at rest and during activity during the first postoperative hour (*p* < 0.001). Although the Sedation-Agitation Scale score at PACU admission was significantly lower in the sufentanil group (*p* < 0.001), the overall recovery profiles, including time to extubation, PACU stay, and QoR-40 scores on POD 1, were comparable between the groups. **Conclusions:** TCI-based sufentanil and remifentanil in TIVA with remimazolam showed similar overall analgesic efficacies and recovery outcomes after laparoscopic subtotal gastrectomy. Both opioid strategies are effective for postoperative pain management, with a slight advantage in immediate postoperative pain control for sufentanil.

## 1. Introduction

The use of opioids during anesthesia plays an important role in recovery and pain management after surgery [1,2,3]. In total intravenous anesthesia (TIVA), opioids are effectively administered through the target-controlled infusion (TCI) method, which ensures rapid achievement and maintenance of target plasma or effect-site concentrations, thereby guaranteeing a stable anesthetic state [4]. Remifentanil, characterized by its rapid onset and short context-sensitive half-life, is particularly well-suited for the TCI approach [5,6]. In contrast, sufentanil, a more potent opioid with a longer-lasting analgesic effect, has a longer context-sensitive half-life. However, its use in TCI helps prevent prolonged tissue accumulation and facilitates quick recovery from anesthesia [7,8]. When combined with propofol, sufentanil-based TCI has been reported to offer more effective postoperative pain management than remifentanil [9,10,11].

Remimazolam is characterized by rapid metabolism, superior hemodynamic stability, and the availability of a specific reversal agent [12,13]. These properties make it an attractive alternative to propofol for TIVA, avoiding injection pain and risks associated with long-term infusion [14,15]. However, research comparing sufentanil with remifentanil is lacking in the context of recovery and postoperative analgesia in TIVA using remimazolam. Because remimazolam, a new anesthetic lacking intrinsic analgesic properties, was used equally in both groups and therefore had minimal influence on postoperative pain perception, a direct comparison between opioids was possible [16]. Accordingly, our study aimed to explore the effects of TCI-based sufentanil versus remifentanil with remimazolam on postoperative pain control and recovery outcomes in patients undergoing laparoscopy-assisted subtotal gastrectomy.

## 2. Material and Methods

### 2.1. Study Populations

The study included adult patients aged 20 years and above who were scheduled to undergo laparoscopic subtotal gastrectomy at Yonsei University Health System, Severance Hospital, Seoul. Participants were required to be classified as having an American Society of Anesthesiologists physical status I–III. Exclusion criteria included patients with emergency or unplanned surgeries, known allergies to the study drugs or components, severe reactions to dextran-40, genetic lactose metabolism disorders, drug or alcohol dependence, acute alcohol intoxication with depressed vital signs, sleep apnea syndrome, uncontrolled medical conditions, unstable psychiatric disorders, inability to use patient-controlled analgesia (PCA), body mass index > 30, and pregnant or potentially pregnant women. The study was approved by the Institutional Review Board and Hospital Research Ethics Committee at Yonsei University Health System in Seoul, Korea (IRB No.: 4-2022-1606) on 13 February 2023. Additionally, the study was officially registered with a clinical trial registry accessible at www.clinicaltrials.gov on 28 February 2023 under the identifier NCT05785234. All subjects voluntarily signed a written informed consent form before being enrolled in the trial.

### 2.2. Study Design

This was a prospective randomized controlled trial designed to evaluate the effects of sufentanil–remimazolam versus remifentanil–remimazolam on postoperative analgesia and recovery following laparoscopic subtotal gastrectomy. The study was conducted following IRB approval between March 2023 and December 2023, with a planned sample size of 66 patients randomly divided into remifentanil (*n* = 33) and sufentanil (*n* = 33) groups. A random allocation sequence was generated using www.random.org (accessed on 20 February 2023). This study employed a double-blind design regarding patients and outcome assessors. Patients were blinded to their group allocation. Postoperative outcome assessors, including PACU and ward nurses, were blinded to the study group assignment. However, the attending anesthesiologist could not be blinded because active management of different TCI models and specific bridging protocols were required.

In the sufentanil group, participants received TIVA with sufentanil–remimazolam. Anesthesia induction was initiated with remimazolam (Byfavo injection; Hana Pharm. Co., Ltd., Seoul, Republic of Korea) at 6 mg/kg/h, followed by a maintenance infusion of 1–2 mg/kg/h after loss of consciousness. Sufentanil (Sufental Injection, BC World Pharm. Co., Ltd., Seoul, Republic of Korea) was administered using the Gepts model for TCI, starting with 0.5 ng/mL for intubation and subsequently adjusted between 0.2 and 0.6 ng/mL based on patient response. In the remifentanil group, the patients were administered TIVA with remifentanil and remimazolam. Induction involved remimazolam at 6 mg/kg/h, followed by a continuous infusion of 1–2 mg/kg/h. Remifentanil was delivered using the Minto model for TCI, with an initial dose of 4 ng/mL for intubation, followed by a maintenance range of 2–6 ng/mL, tailored to individual patient conditions. For both groups, dosages were adjusted according to the patients’ hemodynamic responses and surgical stimuli, ensuring optimal anesthesia depth and analgesia throughout the surgical procedure.

### 2.3. Anesthetic Management

General anesthesia was administered in accordance with Severance Hospital’s standard clinical procedures. Upon arrival in the operating room, patients underwent comprehensive monitoring, including blood pressure assessments, electrocardiograms, oxygen saturation examination, electromyography (Tetragraph^®^, Senzime, Uppsala, Sweden), Patient State Index (PSI; SedLine^®^, Masimo Corp., Irvine, CA, USA), and the Analgesia Nociception Index (ANI; ANI^®^, Masimo Corp., Irvine, CA, USA). Patients were preoxygenated with a 100% oxygen mask for 3 min and received an anticholinergic agent, glycopyrrolate (0.1 mg), as premedication. Anesthesia induction followed a protocol specific to the assigned group: the sufentanil group received a protocol based on sufentanil–remimazolam, whereas the remifentanil group was managed with remifentanil–remimazolam. Upon confirmation of unconsciousness, marked by the absence of verbal responsiveness and eyelash reflexes, neuromuscular blockade was achieved using 0.6 mg/kg intravenous rocuronium. The adequacy of muscle relaxation was verified by using Tetragraph^®^ to ensure a train-of-four count of zero before intubation. Mechanical ventilation was initiated with a fractional inspired oxygen of 0.5, a tidal volume of 6–8 mL/kg, and a positive end-expiratory pressure of 5 cmH_2_O, and an inspiratory–to–expiratory ratio of 1:2. The respiratory rate was adjusted to maintain end-tidal CO_2_ at 35–42 mmHg. Throughout the surgery, anesthesia maintenance was strictly titrated to maintain the PSI within 40–60 and ANI between 50 and 70. The target for hemodynamic stability was to maintain both mean arterial pressure (MAP) and heart rate (HR) within a 20% variance of their respective baseline measurements, with hypotension management using additional fluid administration, ephedrine (4–6 mg), or phenylephrine (20 µg), as needed. At the end of the surgery, we administered sugammadex (2–4 mg/kg) to reverse muscle relaxation, guided by train-of-four count stimulation. Remimazolam infusion was discontinued at the end of the procedure, and flumazenil (Flunil^®^ Bukwang Pharm Co., Ltd., Seoul, Republic of Korea) 0.2–0.3 mg was administered for the reversal of sedation. Additional doses of flumazenil were given as needed, ensuring that the total dose did not exceed 0.5 mg, to facilitate complete awakening and recovery of respiratory function before extubation and transfer to the post-anesthesia care unit (PACU).

### 2.4. Postoperative Pain Management

Prior to the completion of surgical suturing, all patients were administered 1 g of acetaminophen intravenously (IV) for pain management. For antiemetic prophylaxis, 0.3 mg of IV ramosetron (Nasea^®^, Astellas Pharma Korea, Seoul, Republic of Korea) was also injected. To account for the distinct pharmacokinetic profiles of the two opioids, different transition strategies were employed. In the remifentanil group, considering its rapid offset and the risk of immediate postoperative pain, patients received a dose of 1 µg/kg of IV fentanyl during subcutaneous suturing. Remifentanil TCI was maintained at a concentration of 1–2 ng/mL until extubation. In the sufentanil group, sufentanil TCI was maintained at an effective concentration of 0.1–0.2 ng/mL until extubation to utilize its residual analgesic effect for a smooth transition, without additional bolus administration. These target concentrations for both groups were titrated to maintain the ANI between 50 and 70, ensuring equipotent antinociceptive depth prior to extubation.

Postoperative analgesia was administered using an IV-PCA pump (WooYoung Medical, Seoul, Republic of Korea) filled with a 250 mL solution containing fentanyl (30 µg/kg) and ramosetron (0.3 mg) in 0.9% saline. We programmed the device with a basal infusion rate of 0.1 mL/min (providing 0.0024 µg/kg/min of fentanyl), a bolus volume of 2 mL (0.24 µg/kg), and a lockout period of 10 min. Before surgery, in the waiting area, patients received instruction on operating the PCA machine and reporting pain severity using the Numeric Pain Rating Scale (NPRS, 0–10). Upon transfer to the PACU and as the patients emerged from anesthesia, pain intensity was assessed using the NPRS. The assessment was conducted by recovery nurses who did not participate in the study. For pain with an NPRS score of 4 or higher, 0.5–1 µg/kg fentanyl was administered as a rescue analgesic. In the ward, patients received additional instructions on how to use the PCA device effectively, specifically being encouraged to activate the bolus button for pain levels exceeding an NPRS score of 3. For patients experiencing persistent pain with NPRS scores of 4 or higher refractory to PCA boluses, we provided additional analgesia using 50 mg of IV tramadol (Tridol^®^, Yuhan Co., Seoul, Republic of Korea).

### 2.5. Outcomes

The primary outcome of this study was the total fentanyl dose administered within the first 24 h postoperatively. Secondary outcomes included pain intensity at rest and during movement, measured using the NPRS at specified time points: immediately post-surgery (0–1 h) and at 6, 12, 18, and 24 h after surgery. Anesthesia recovery characteristics were assessed, including time to extubation, PACU length of stay, and quality of recovery on postoperative day 1 (POD1) using the Quality of Recovery-40 (QoR-40) questionnaire.

### 2.6. Data Collection

Patient characteristics including age, body mass index, previous episodes of postoperative nausea and vomiting and/or motion sickness, smoking history, and previous exposure to chemotherapy or radiotherapy were systematically collected from medical records and preoperative interviews. The American Society of Anesthesiologists physical status classification was assessed by experienced anesthesiologists. The risk of PONV was assessed using the Apfel score (range 0–4), based on female gender, non-smoking status, history of PONV or motion sickness, and postoperative opioid use.

Intraoperative hemodynamic parameters, including MAP, HR, pulse pressure variation (PPV), and cardiac index (CI), were measured at predefined time points: prior to induction, post-intubation, at the onset of CO_2_ pneumoperitoneum, and at regular 30 min intervals throughout surgery.

Upon admission to the PACU, detailed assessments were conducted to document pain profile, Riker Sedation-Agitation Scale, incidence of nausea and vomiting, and NPRS scores. Intraoperative and surgical details, such as the presence of adhesions, omentectomy, estimated blood loss, total fluid intake, urine output, duration of anesthesia, and intraoperative doses of anesthetic agents and vasoactive drugs, were systematically documented.

On POD1, quality of recovery was evaluated using the QoR-40 questionnaire, which assesses five domains: physical comfort, physical independence, emotional status, psychological support, and pain. We also evaluated patient satisfaction using a 4-point scale (1, very unsatisfied; 4, very satisfied), and the incidence of postoperative nausea and vomiting.

### 2.7. Statistical Analysis

Based on a previous study comparing sufentanil and remifentanil, which reported a total morphine consumption of 37 mg (standard deviation of 20 mg) in the sufentanil group and 56 mg (standard deviation of 29 mg) in the remifentanil group over 24 h postoperatively, the required sample size for each group was calculated [9]. With an alpha level of 0.05 and a power of 80%, the minimum required sample size was determined as 29 patients per group, totaling 58 patients. To account for a potential 10% attrition rate, the final sample size was adjusted to 33 subjects per arm, resulting in a total recruitment target of 66 patients.

Continuous variables were reported as means and standard deviations, and compared via Student’s *t*-test. Categorical variables were expressed as frequencies (*n*) and percentages (%). Group comparisons were performed using the chi-square test or Fisher’s exact test, as appropriate. For repeated-measures variables, we utilized a linear mixed model to account for correlations within subjects over time, assuming an unstructured covariance structure. Post hoc adjustments for multiple comparisons were made using the Bonferroni method. Statistical significance was defined as a *p*-value < 0.05. All statistical computations were executed using the SAS software (version 9.4; SAS Institute Inc., Cary, NC, USA).

## 3. Results

**Figure 1:**
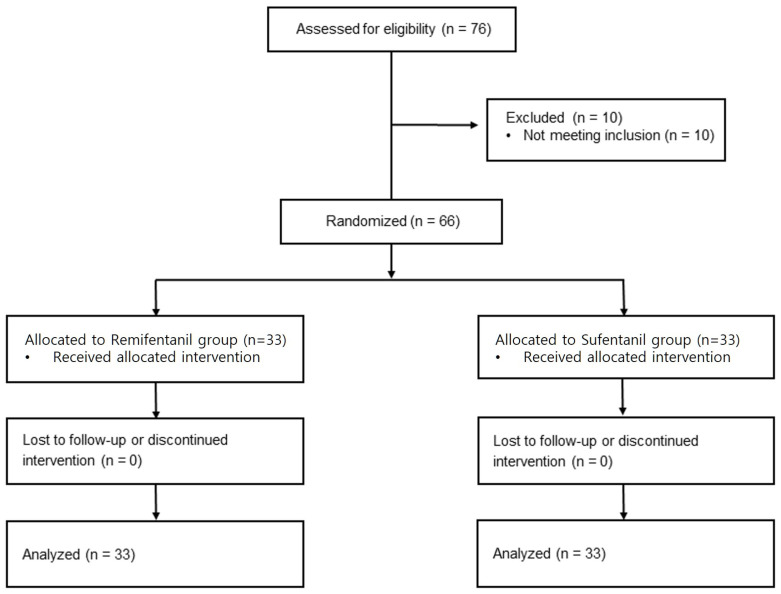
Consolidated Standards of Reporting Trials flow diagram.

Sixty-six patients undergoing laparoscopy-assisted subtotal gastrectomy underwent an eligibility assessment and were randomized to two groups. The final dataset comprised all 66 participants without any exclusions (Figure 1). There were no protocol deviations, missing data points, or early discontinuations of the PCA device among the enrolled participants.

Patient characteristics are described in Table 1 and Table 2 presents the intraoperative and surgical characteristics, which were comparable between the two groups.

**Figure 2:**
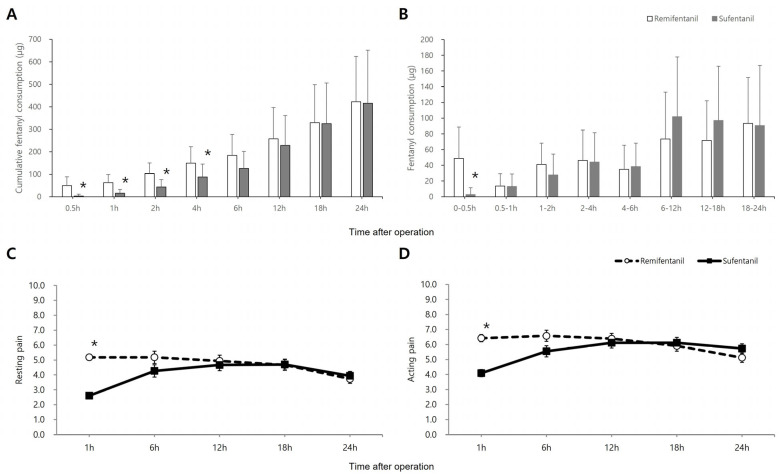
Pain related profile: (**A**) Cumulative dose of fentanyl consumption and (**B**) fentanyl consumption during the 24 h postoperative period. Plots (**C**,**D**) illustrate the Numeric Pain Rating Scale (NPRS) scores for pain at rest and pain during movement, respectively, over the 24 h postoperative course. Data points in (**A**,**B**) represent the mean ± standard deviation. Data points in (**C**,**D**) represent the mean ± standard error. * Bonferroni-corrected *p* < 0.05 versus the control group.

Figure 2A displays the cumulative dose of fentanyl consumed, whereas Figure 2B illustrates fentanyl administration during the 24 h following surgery. The cumulative fentanyl consumption over the 24 h postoperative period was comparable between the two groups (422.39 ± 201.61 µg in the remifentanil group vs. 415.55 ± 236.64 µg in the sufentanil group; Mean Difference, 6.84 µg; 95% CI, −101.27 to 114.95; *p* = 0.900). Significant differences between the groups were noted in cumulative fentanyl consumption at 0.5, 1, 2, and 4 h (Bonferroni-corrected *p* < 0.001, < 0.001, < 0.001, and 0.003, respectively) (Figure 2A). However, the sufentanil group required significantly less fentanyl only during the immediate postoperative period (0–0.5 h, Bonferroni-corrected *p* < 0.001) (Figure 2B) compared to the remifentanil group. Figure 2C,D show the resting and active NPRS scores, respectively. NPRS scores for both resting and active pain were significantly lower in the sufentanil group compared to the remifentanil group during the first postoperative hour (all Bonferroni-corrected *p* < 0.001).

**Figure 3:**
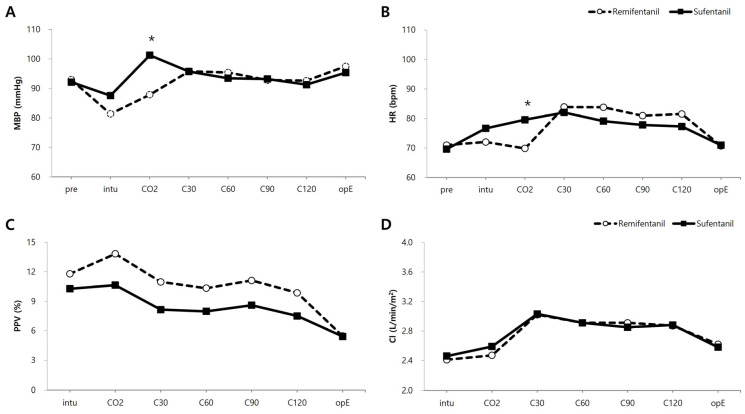
Intraoperative hemodynamics: Intraoperative hemodynamic changes are depicted for (**A**) mean blood pressure (MBP), (**B**) heart rate (HR), (**C**) pulse pressure variation (PPV), and (**D**) cardiac index (CI). The figures show the average values calculated using the linear mixed models, accompanied by standard errors. These measurements were taken at several time points: prior to induction (Pre); 10 min post-intubation (intu); at the onset of CO_2_ pneumoperitoneum and when positioning the patient head-up (CO_2_); 30 min (C30), 60 min (C60), 90 min (C90), and 120 min (C120) after starting CO_2_ pneumoperitoneum; and at the end of the operation (OpE). Vasoactive agents (ephedrine and phenylephrine) were administered as needed (PRN) to manage hemodynamic fluctuations, rather than at fixed time points. The overall significance of the time-related changes was *p* < 0.001. * Bonferroni-corrected *p* < 0.05 between the two groups.

Figure 3 illustrates the intraoperative hemodynamic profiles. We noted significant differences in the MAP and HR between the sufentanil and remifentanil TCI groups during CO_2_ insufflation and head-up reverse Trendelenburg positioning. However, no differences were noted between the groups at other time points regarding changes in MAP, HR, PPV, and CI.

Postoperative pain profiles and primary outcomes are summarized in Table 3. During the PACU period, the sufentanil group exhibited significantly lower peak NPRS scores, required fewer rescue analgesics, and consumed less total fentanyl compared to the remifentanil group (2.61 vs. 5.24, *p* < 0.001; 2 [6%] vs. 26 [79%], *p* < 0.001; and 0 [0–0] µg vs. 40 [25–50] µg, *p* < 0.001, respectively).

Secondary recovery outcomes are presented in Table 4. The time required for extubation was comparable between the two groups. In the PACU, the Riker Sedation-Agitation Scale score at admission was significantly reduced in the sufentanil group compared with the remifentanil group (3.52 ± 0.51 vs. 3.91 ± 0.29, *p* < 0.001); however, the duration of PACU stay was comparable between the two groups. Regarding quality of recovery, we observed no significant intergroup differences in QoR-40 scores on POD 1. Furthermore, no differences in other aspects of the recovery profiles were noted between the two groups.

## 4. Discussion

This prospective, double-blind, randomized controlled trial evaluated the efficacy of intraoperative TIVA using sufentanil–remimazolam or remifentanil–remimazolam on postoperative analgesia and recovery in patients undergoing laparoscopy-assisted subtotal gastrectomy. Our findings revealed no significant difference in cumulative fentanyl consumption during the postoperative 24 h period between the two groups. However, patients receiving sufentanil required significantly less fentanyl during the immediate postoperative period (0–0.5 h), and cumulative fentanyl consumption was also reduced during the first 4 h. In addition, NPRS scores at rest and on activity were lower in the sufentanil group during the first hour after surgery. Specifically, the interval analysis indicates that the difference in cumulative consumption observed up to 4 h was primarily driven by the marked reduction in opioid requirement during the immediate postoperative phase (0–0.5 h). Based on these findings, TCI-based sufentanil TIVA with remimazolam appears to provide better postoperative analgesic effects during the early postoperative period compared to TCI-based remifentanil TIVA with remimazolam with a fentanyl bolus (1 µg/kg).

Previous studies comparing sufentanil and remifentanil in propofol-based TIVA reported that sufentanil can reduce postoperative analgesic requirements [9,10,17,18]. Derrode et al. reported significantly lower 24 h cumulative morphine consumption with sufentanil TCI (0.25 ng/mL effect-site concentration at extubation) compared to remifentanil TCI combined with a morphine bolus (0.15 mg/kg) in patients undergoing major abdominal surgery [9]. While our findings are consistent with this result, the duration of sufentanil’s residual analgesic effect was shorter in this study. This difference could be explained by the lower effect-site concentration of sufentanil (0.1–0.2 ng/mL at extubation) used in our laparoscopic surgery protocol, whereas Derrode’s study involved open surgery and utilized a morphine PCA regimen, unlike the fentanyl PCA used in our study. Similarly to our findings, Bidgoli et al. reported lower postoperative piritramide consumption with sufentanil TCI (0.3 ng/mL effect-site concentration, discontinued at skin closure) than with remifentanil TCI during the first 4 h postoperatively in morbidly obese patients undergoing laparoscopic gastroplasty [18]. Our research is significant as we compared sufentanil and remifentanil in remimazolam-based TIVA, addressing a data gap in the literature that has primarily focused on propofol-based regimens.

In the present study, we utilized digital monitoring, specifically the PSI and the ANI, to ensure precise and patient-specific anesthetic delivery. The PSI is a high-resolution, 4-channel electroencephalography derived index used to monitor the hypnotic effects of general anesthetics. Recent comparative studies have demonstrated that PSI serves as a reliable hypnotic indicator during remimazolam sedation [19]. Furthermore, we employed the ANI, which quantifies parasympathetic activity based on the high-frequency components of heart rate variability. Evidence indicates that the ANI reliably reflects noxious stimulation even during remimazolam-based anesthesia [16]. However, despite precise anesthetic titration guided by these monitors, significant differences were observed in immediate postoperative pain scores and rescue analgesic requirements. This suggests that opioid pharmacokinetics—sufentanil’s sustained effect versus remifentanil’s rapid offset—more strongly influence early recovery (0–4 h) than the monitoring method itself.

The clinical outcomes observed in this study corroborate the theoretical expectations based on the distinct pharmacokinetic profiles of the two opioids. The residual analgesic effects of sufentanil reported in previous studies are partly attributed to its pharmacokinetic features. Sufentanil, a piperidine derivative with an N-4 substitution compared to fentanyl, exhibits high affinity for μ-opioid receptors and is approximately 6–10 times more potent than fentanyl [20]. This enhanced potency makes sufentanil well-suited for maintaining both intraoperative and postoperative analgesia. However, sufentanil has a longer context-sensitive half-life than remifentanil (34 versus 3.6 min, respectively, after a 200 min infusion) [21]. Remifentanil, a short-acting μ-opioid receptor agonist, maintains a constant context-sensitive half-life (3–5 min), regardless of infusion duration [22]. In contrast, the context-sensitive half-life of sufentanil increases with prolonged infusion. Despite this pharmacokinetic difference, the TCI mode optimizes opioid administration by targeting blood or effect-site concentrations, ensuring accurate anesthesia depth, minimizing overdose risks, limiting long-acting opioid accumulation, and enabling rapid recovery from anesthesia [23]. We administered remifentanil using the Minto model and sufentanil using the Gepts model for TCI. Our data demonstrated that sufentanil TCI provided adequate pain control in the immediate postoperative period compared to remifentanil TCI with a fentanyl bolus, without delaying extubation or causing respiratory depression.

Furthermore, considering that remifentanil is associated with acute opioid tolerance and hyperalgesia, the significantly higher opioid requirement in the remifentanil group during the immediate postoperative period may be partly attributed to opioid-induced hyperalgesia associated with remifentanil use [3,24]. Our findings suggest that the tapering strategy of sufentanil effectively mitigated these rebound effects. Although the Riker Sedation-Agitation Scale score was significantly lower in the sufentanil group (3.52 ± 0.51) than in the remifentanil group (3.91 ± 0.29) upon immediate PACU admission, this difference was transient and clinically insignificant. The absence of any significant difference in PACU stay duration further suggests that sufentanil TCI did not adversely affect postoperative sedation or agitation.

In contrast, the clinical relevance of the early analgesic efficacy provided by sufentanil is substantial. Although the advantage in NPRS scores was limited to the first hour, this period represents the critical transition from anesthesia to recovery. Effective pain control during this phase is important for patient safety and comfort. Indeed, the requirement for rescue analgesics in the PACU was dramatically lower in the sufentanil group (6%) compared to the remifentanil group (79%). This indicates that the sufentanil regimen provided superior analgesic coverage during the critical transition phase, significantly enhancing patient comfort and reducing the need for immediate nursing interventions.

CO_2_ insufflation and head-up reverse Trendelenburg positioning are known to potentially induce hemodynamic instability by decreasing cardiac output [25]. Furthermore, CO2 pneumoperitoneum itself constitutes a major nociceptive stimulus, inducing significant sympathetic activation that results in cardiovascular effects such as tachycardia and hypertension [26]. During these phases, we noted significant differences in MAP and HR between the sufentanil and remifentanil TCI groups. These hemodynamic differences may be explained by the different pharmacokinetic properties and antinociceptive profiles of sufentanil and remifentanil during CO_2_ insufflation [27]. The observed transient hemodynamic instability in the remifentanil group during this high-nociceptive period supports the clinical utility of intraoperative digital monitoring, such as the ANI, for ensuring stable antinociception. We regulated opioids under the guidance of PSI and ANI, increasing sufentanil up to 0.5–0.6 ng/mL and remifentanil up to 5–6 ng/mL during surgery. Thirty minutes before the end of surgery, the target effect-site concentrations were lowered to achieve adequate analgesia while maintaining spontaneous breathing. Importantly, while these hemodynamic differences were statistically significant, they did not reflect clinically detrimental instability, as evidenced by comparable requirements for vasoactive drugs (ephedrine, phenylephrine, and nicardipine) between groups (Table 2), indicating that both opioids can be safely managed with appropriate monitoring and titration.

Regarding the choice of hypnotic, Lee et al. reported that remimazolam combined with remifentanil could maintain MAP more effectively than sevoflurane by mitigating the effects of CO_2_ insufflation and reverse Trendelenburg positioning [28]. While they observed higher CI, MAP, and HR in the remimazolam group, our study found no difference in CI between the sufentanil–remimazolam and remifentanil–remimazolam groups, suggesting that cardiac output is preserved regardless of the opioid used in this setting. Moreover, both groups showed similar MAP, HR, PPV, and CI at other time points. This overall hemodynamic stability is consistent with Vasian et al., who demonstrated stable hemodynamics with both opioids during propofol-based open colorectal surgery [27]. Notably, their observation that sufentanil required fewer dose adjustments and produced smaller decreases in MAP and HR during induction aligns with our finding of superior hemodynamic stability with sufentanil during acute nociceptive challenges.

The QoR-40 on POD 1, which is a reliable objective measure of the quality of recovery following anesthesia and surgery [29], showed no differences between both groups across all five dimensions assessed in the questionnaire. Additionally, no significant differences were observed between the groups in the incidence of postoperative nausea and vomiting, patient satisfaction, or length of postoperative hospital stay. The results of the present study correspond well with previous findings. According to De Baerdemaeker et al., the type of opioid, whether remifentanil or sufentanil TCI, has minimal impact on postoperative recovery [30]. Regarding PONV, recent studies suggest that remimazolam may reduce the incidence of nausea compared to volatile agents or other hypnotics [31,32]. Additionally, the impact of patient factors such as obesity on PONV remains controversial, but recent studies suggest it is not a major independent risk factor [33,34]. In our study, although we did not perform a stratified analysis by BMI, the overall simplified incidence of PONV showed no significant difference between groups, which may be partly attributed to the anti-emetic properties of the TIVA regimen itself.

We acknowledge several limitations in this research. First, the study was conducted at a single institution with a limited sample size. Second, the evaluation of opioid consumption and pain intensity was confined to the initial 24 h postoperative period, without assessment of longer-term outcomes such as chronic pain. Third, this study did not compare different opioid concentrations at extubation but rather employed clinically low-dose effect-site concentrations, due to the prolonged recovery time associated with the pharmacokinetic profile of sufentanil. Future studies should aim to determine the optimal sufentanil concentration that maximizes analgesic effectiveness without compromising recovery. Fourth, we did not collect data on patients’ baseline pain history or chronic opioid use, which could influence postoperative analgesic requirements. Additionally, we did not perform a cost-effectiveness analysis comparing the two opioid regimens. Fifth, although PSI and ANI were utilized as real-time guides for anesthetic titration, continuous data logging was not performed for post hoc analysis. Sixth, the study design compared sufentanil TCI tapering against remifentanil TCI combined with a specific fentanyl bridging bolus (1 µg/kg). Therefore, the superior immediate analgesia observed in the sufentanil group might be partlyh attributable to the specific bridging protocol rather than solely the intrinsic pharmacokinetic properties of sufentanil. A higher bolus dose in the remifentanil group might have yielded different results. Seventh, the attending anesthesiologist could not be blinded to the group allocation due to the necessity of managing different drug administration protocols. However, to minimize potential bias, all postoperative data collection and outcome assessments were performed by blinded personnel. Finally, although we assessed general recovery outcomes, specific gastrointestinal recovery metrics such as time to first flatus or defecation, as well as specific postoperative pulmonary complications, were not prospectively recorded. Despite these limitations, this study is clinically significant as it contributes to our understanding of remimazolam, a novel ultra-short-acting benzodiazepine, and its role in TIVA for patients undergoing laparoscopy-assisted subtotal gastrectomy, particularly in terms of postoperative analgesia and recovery. Future studies focusing on these functional recovery indices in gastric surgery are warranted.

## 5. Conclusions

This study compared TCI-based sufentanil and remifentanil for remimazolam TIVA during laparoscopic subtotal gastrectomy. The sufentanil group demonstrated superior early postoperative pain control. Although no significant difference was observed in cumulative fentanyl consumption over 24 h, the sufentanil group required significantly less fentanyl during the immediate postoperative period (0–0.5 h) and reported lower pain scores during the first hour. Furthermore, the remifentanil group showed transient hemodynamic instability during CO_2_ insufflation. No significant differences were found in recovery outcomes beyond the immediate postoperative period, including QoR-40 scores on POD1. These findings suggest that both opioid strategies provide effective postoperative pain management with comparable overall recovery, although sufentanil offers a distinct advantage in immediate postoperative pain control and hemodynamic stability. The observed hemodynamic instability during high nociceptive stimulation underscores the utility of digital monitoring tools such as PSI and ANI for optimizing intraoperative anesthetic management. Further studies are required to explore long-term benefits and broader clinical applications.

## Figures and Tables

**Table 1:** Preoperative demographic characteristics.

Variables	Remifentanil Group (*n* = 33)			Sufentanil Group (*n* = 33)			*p*
Age, years	61.85	±	9.42	58.36	±	10.24	0.155
Sex (Male/Female)	17 (51.5)	/	16 (48.5)	20 (60.6)	/	13 (39.4)	0.457
BMI, kg/m^2^	22.56	±	3.17	22.64	±	3.11	0.915
ASA physical status							0.091
1	6		[18.18]	9		[27.27]	
2	17		[51.52]	21		[63.64]	
3	10		[30.30]	3		[9.09]	
History of PONV and/or motion sickness	10		[30.30]	8		[24.24]	0.580
Smoking history							0.217
Nonsmoker	23		[69.70]	19		[57.58]	
Ex-smoker	6		[18.18]	12		[36.36]	
Current smoker	4		[12.12]	2		[6.06]	
Apfel score	2.62	±	1.02	2.58	±	0.87	0.698

Notes: Descriptive statistics such as mean and standard deviation were calculated for continuous variables, and frequencies (*n*) and [percentages (%)] were reported for categorical variables. Abbreviations: BMI, body mass index; ASA, American Society of Anesthesiologists; PONV, postoperative nausea vomiting.

**Table 2:** Intraoperative and surgical characteristics.

Variables	Remifentanil Group (*n* = 33)			Sufentanil Group (*n* = 33)			*p*	
Time to loss of consciousness, sec	140.39	±	32.59	153.03	±	38.21	0.153	
Anesthesia time, min	221.42	±	57.15	207.06	±	39.78	0.241	
Operation time, min	184.18	±	57.65	170.12	±	40.97	0.258	
Total fluid intake, mL	1677.27	±	395.70	1554.55	±	403.18	0.217	
Blood loss, mL	75.82	±	68.86	51.18	±	53.16	0.109	
Urine output, mL	287.36	±	167.18	291.97	±	307.91	0.940	
Intraoperative administered anesthetics								
Remifentanil, µg	1375.36	±	524.20				-	
Sufentanil, µg				59.86	±	22.08	-	
Remimazolam, µg	286.41	±	97.59	271.39	±	87.11	0.512	
Intraoperative vasoactive drugs								
Nicardipine, *n*	1		[3.03]	2		[6.06]	>0.999	
Nicardipine, µg	45.45	±	261.12	36.36	±	163.59	0.866	
Phenylephrine, *n*	7		[21.21]	3		[9.09]	0.170	
Phenylephrine, µg	209.03	±	916.36	186.67	±	706.48	0.912	
Ephedrine, *n*	7		[21.21]	5		[15.15]	0.523	
Ephedrine, mg	1.58	±	3.73	0.97	±	2.60	0.448	
Operative findings								
Intraoperative adhesion								
(none/mild/moderate/severe)							0.708	
None	5		[15.15]	7		[21.21]		
Mild	23		[69.70]	24		[72.73]		
Moderate	3		[9.09]	1		[3.03]		
Severe	2		[6.06]	1		[3.03]		
Omentectomy							0.492	
(not done/partial/complete)								
not done	0		[0]	1		[3.03]		
Partial	33		[100]	31		[93.94]		
Complete	0		[0]	1		[3.03]		

Notes: Values are presented as mean ± standard deviation (SD) for continuous data and as number [percentage (%)] for categorical data.

**Table 3:** Primary Outcomes: Pain & Opioid Consumption.

Variables	Remifentanil Group (*n* = 33)			Sufentanil Group (*n* = 33)			*p*
Cumulative Fentanyl Consumption (24 h), µg	422.39	±	201.61	415.55	±	236.64	0.900
Maximal NPRS score (0–10)	5.24	±	1.03	2.61	±	1.17	<0.001
Rescue analgesics, *n*	26		[78.79]	2		[6.06]	<0.001
Rescue analgesics, µg	40		[25–50]	0		[0–0]	<0.001

Notes: Values are presented as mean ± standard deviation (SD) for cumulative consumption and number (%) for categorical variables. Rescue analgesics are presented as median [interquartile range] due to non-normal distribution. Abbreviations: NPRS, Numeric Pain Rating Scale.

**Table 4:** Secondary Outcomes: Recovery & Quality of Life.

Variables	Remifentanil Group (*n* = 33)			Sufentanil Group (*n* = 33)			*p*
Time to extubation, sec	291	±	119	319	±	105	0.318
SAS at emergence (1–7)	3.94	±	0.24	3.85	±	0.36	0.238
Recovery room data							
SAS at PACU admission (1–7)	3.91	±	0.29	3.52	±	0.51	<0.001
Nausea, *n*	2		[6.06]	1		[3.03]	>0.999
Vomiting, *n*	0		[0.00]	0		[0.00]	-
PACU stay, min	61.48	±	25.71	51.73	±	30.96	0.169
QoR-40 on POD1							
Physical comfort	47.12	±	5.78	44.33	±	6.85	0.079
Physical independence	14.82	±	4.86	13.97	±	4.56	0.468
Emotional status	34.79	±	5.77	34.00	±	6.09	0.591
Psychological support	29.30	±	5.76	29.61	±	4.29	0.809
Pain	28.70	±	4.07	27.73	±	4.72	0.375
Global QoR-40 score	154.73	±	18.41	149.64	±	21.04	0.299
PONV on POD1							
Nausea, *n*	9		[27]	17		[52]	0.085
Vomiting, *n*	3		[9]	4		[12]	>0.999
Patient satisfaction score (1–4)	2.85	±	0.71	2.70	±	0.73	0.396
Postoperative hospital stays, days	5.15	±	1.28	5.12	±	0.86	0.910

Notes: Descriptive statistics such as the mean and standard deviation were calculated for continuous variables, frequencies (*n*) and [percentages (%)] were reported for categorical variables. Abbreviations: SAS, Riker Sedation-Agitation Scale; PACU, post-anesthesia care unit; QoR, quality of recovery; POD1, postoperative day 1; PONV, postoperative nausea and vomiting; Riker Sedation-Agitation Scale: 1 = unarousable, 2 = very sedated, 3 = sedated, 4 = calm and cooperative, 5 = agitated, 6 = very agitated, and 7 = dangerous.

## Data Availability

The datasets used and/or analyzed during the current study are available from the corresponding author on reasonable request.

## Abbreviations

The following abbreviations are used in this manuscript:

TIVA	Total intravenous anesthesia
TCI	Target-controlled infusion
PCA	Patient-controlled analgesia
IRB	Institutional review boar
PSI	Patient State Index
ANI	Analgesia Nociception Index
MAP	Mean arterial pressure
HR	Heart rate
PACU	Post-anesthesia care unit
IV	Intravenously
NPRS	Numeric Pain Rating Scale
POD1	Postoperative day 1
QoR-40	Quality of Recovery-40
PPV	Pulse pressure variation
CI	Cardiac index
